# Respiratory symptoms and illnesses among brick kiln workers: a cross sectional study from rural districts of Pakistan

**DOI:** 10.1186/1471-2458-12-999

**Published:** 2012-11-20

**Authors:** Shiraz Shaikh, Asaad Ahmed Nafees, Vikash Khetpal, Abid Ali Jamali, Abdul Manan Arain, Akram Yousuf

**Affiliations:** 1 Department of Community Health Sciences, Aga Khan University, Stadium Road, PO Box 3500, Karachi, 74800, Pakistan; 2 Shaheed Mohtarma Benazir Bhutto University, Larkana, Pakistan

**Keywords:** Brick kiln workers, Pakistan, Respiratory symptoms and illnesses, Chronic bronchitis, Asthma

## Abstract

**Background:**

Occupational risk factors are one of the major causes of respiratory illnesses and symptoms, and account for 13% of chronic obstructive pulmonary disease and 11% of asthma worldwide. Majority of brick kilns in Pakistan use wood and coal for baking the bricks which makes the brick kiln workers susceptible to high exposure of air pollution**.** This study was designed to describe frequency of chronic respiratory symptoms and illnesses and study the association between these symptoms and different types of work.

**Methods:**

This was a questionnaire based cross sectional survey conducted among the brick kiln workers in Larkana and Dadu districts, Sindh, Pakistan. A total of 340 adult men were assessed using translated version of the American Thoracic Society Division of Lung Disease (ATS-DLD) questionnaire. Logistic regression analysis was done to determine the relationship between various socio-demographic and occupational factors (age, education, type of work, number of years at work, smoking status), and the respiratory symptoms and illnesses (chronic cough, chronic phlegm, wheeze, Chronic Bronchitis and asthma).

**Results:**

Results of the study show that 22.4% workers had chronic cough while 21.2% reported chronic phlegm. 13.8% had two or more attacks of shortness of breath with wheezing. 17.1% workers were suffering from Chronic Bronchitis while 8.2% reported physician diagnosed asthma. Amongst the non-smoking workers 8.9% had Chronic Bronchitis. Multivariate analysis found that workers involved in brick baking were more likely to have Chronic Bronchitis (OR= 3.7, 95% CI 1.1-11.6, p=<0.05) and asthma (OR= 3.9, 95% CI 1.01-15.5, p=<0.05) compared to those involved in carriage and placement work.

**Conclusion:**

A high frequency of respiratory symptoms and illnesses was observed among brick kiln workers. Age, nature of work and smoking were strong predictors of developing these symptoms and illnesses.

## Background

Brick manufacturing involves three main steps: clay shaping with water (molding), drying with solar energy and firing with fuel (baking). Workers at brick kiln may be involved in carrying the clay dust and bricks, molding or baking. Although all the workers are exposed to dust and smoke, molders are more likely to be directly exposed to dust and bakers have more proximal exposure to smoke. Smoke and dust from brick kilns is an important cause of air pollution [1]. Clay dust contains a mixture of inorganic compounds including free silica, iron oxide, lime, magnesium carbonate, alkalis, calcium carbonate, calcium sulfate, sodium chloride and varying amounts of organic materials while burning of biomass fuels increase the exposure to gases including sulphur dioxide, hydrogen sulphide, carbon dioxide and carbon monoxide and particulate air pollutants. Next to smoking, occupational risk factors are a major cause of chronic respiratory illnesses and account for 13% of COPD, 11% of asthma and almost all cases of silicosis, asbestosis and pneumoconiosis worldwide [2]. A review of the literature shows that workers from different occupations exposed to dust and smoke including brick kiln workers are at a higher risk of developing chronic respiratory symptoms and illnesses [3]. Besides environmental exposures, occupational factors also play an important role in affecting the health of the employees. Evidence suggests that factors like length of job, lack of protective equipment, type of work and type of burning fuel is associated with respiratory illnesses in different occupations [4-6].

Little information is available regarding respiratory health of brick kiln workers in developing countries. A study done on brick manufacturing workers in Croatia shows that there is a significantly higher prevalence of respiratory symptoms such as chronic cough (31.8%), chronic phlegm (26.2%) and chest tightness (24%) in exposed workers as compared to control workers (20.1%, 18.1%, 0%) respectively [7].

In Pakistan, a significant number of people are working in brick kilns in the outskirts of towns and cities, contributing greatly to construction sector of the country. Majority of brick kilns in Pakistan use wood and coal for baking the bricks which makes the brick kiln workers susceptible to high exposure of air pollution and its adverse health effects**.** It has been previously estimated that exposure to wood smoke is associated with a 70% increased risk of having chronic obstructive pulmonary disease [8]. These kilns range from small kilns with only 8–10 workers to large ones employing more than 100 workers. Majority of brick kiln are open air Bull Trench Kilns situated outdoors. Particulate pollution at these kilns is 7–8 times more than other types of kilns including Vertical shaft Brick Kilns and Fixed chimney [9]. These air pollutants after inhalation incite inflammation and release of oxygen radicals leading to local tissue injury and pulmonary distress [10].

This study was designed to describe frequency of chronic respiratory symptoms and illnesses and study the association between these symptoms and different types of work (Carriage, Baking, Molding) and length of job.

## Methods

### Study design and population

It was a cross-sectional survey conducted in the outskirts of Larkana and Dadu Districts of the province of Sind, Pakistan during the months of April and May 2011. Unlike other parts of Pakistan where women participate in brick making along with men, work at brick kilns in the outskirts of these towns is carried out almost exclusively by men. Sample size was calculated using the ‘sample size determination in health studies’ software of World Health Organization. Prevalence of respiratory symptoms among brick kiln workers in Croatia [7] was used to calculate the sample size. At confidence level of 95% and bound on the error of 5%, the highest sample size was 335 taking the prevalence of chronic cough 31.8%.

### Selection of study participants

Non-probability, convenience sampling technique was used to select brick kilns and workers due to lack of availability of list of registered brick kilns. Brick kilns within the outskirts of districts Larkana and Dadu were approached and research was undertaken in the kilns where owners gave permission. The workers were selected at the discretion of the study team and kiln owners were not involved in the selection process. Data was collected on weekdays except Sunday. 240 brick kiln workers were interviewed in the outskirts of district Dadu in more than 20 brick kilns while100 brick kiln workers were interviewed in 10 brick kilns in district Larkana. We included all male brick kiln workers above 18 years of age having worked in the kilns for at least 5 years. Written, informed consent was sought from all participating workers before inclusion in the study. The workers were generally cooperative and no refusals were observed.

### Interviews

The data was collected by four 4^th^ year medical students who were given a comprehensive training in which all the details of the research study along with the questionnaire were explained. Quality of data was assessed through random checks by the Principal Investigator. Field testing of the final questionnaire was done before starting the formal data collection. 20 forms from 1or 2 brick kilns were filled daily i-e 5 forms from each student. Every day, these forms were checked for completeness and incomplete forms were excluded. Structured questionnaire was used to obtain detailed information on socio-demographic characteristics and occupational factors. History of chronic respiratory symptoms and illnesses was obtained by using American Thoracic Society Division of Lung Disease questionnaire (ATS-DLD-78A). The questionnaire was reviewed by public health experts for the content of the questions and then translated into the local language. Data was entered in Microsoft Excel software in the form of numeric codes assigned to different variables. It was entered twice and consistency between 2 data sets was checked. The entered data was also verified by cross validating it with 10 randomly picked forms with the hard copies of the data sets.

### Operational definitions of the study outcomes (adopted from ATS guidelines)

***Chronic Cough*** was defined as cough as much as 4–6 times per day occurring for most days of the week (≥5days) for at least three months of the year and for at least two consecutive years. ***Chronic Phlegm*** was classified as sputum expectoration as much as twice a day for most days of the week (≥5days) for at least three months of the year and for at least two consecutive years. ***Dyspnea*** was divided into 5 grades with the following definitions a) Grade 0: No Breathlessness except with strenuous exercise b) Grade 1: Breathlessness when hurrying on the level or walking up a slight hill c) Grade 2: Walking slower than people of the same age on the level because of breathlessness or has to stop for breath when walking at own pace or level d) Grade 3: Stopping for breath after walking about 100 yards (96 meter) or a few minutes on the level e) Grade 4: Too breathless to leave the house or breathless when dressing or undressing. ***Chronic Bronchitis*** was defined as cough and sputum expectoration occurring for most days of the week (≥5days) for at least three months of the year and for at least two consecutive years while ***Asthma*** was classified as at least two or more attacks of shortness of breath with wheezing (whistling sound on expiration) in the past two months with normal breathing in between episodes of shortness of breath or diagnosed asthmatic by a physician.

***Ever smoker*** was defined as more than 20 packs of cigarettes in a lifetime or more than 1 cigarette a day for one year. Ever smoker was further categorized into previous and current smokers. ***Never smoker*** was defined as less than 20 packs of cigarettes in a lifetime or less than 1 cigarette a day in one year.

### Ethical approval

Ethical approval was taken from Ethics Review Committee at Shaheed Mohtarma Benazir Bhutto University, Larkana, Pakistan.

### Statistical analysis

Data was analyzed using software of Statistical Package of Social Sciences (SPSS version 11.5). Descriptive statistics of socio-demographic variables were computed as mean, standard deviation or frequency percentages. Prevalence was estimated for presence of chronic respiratory symptoms and illnesses by calculating the frequency percentages of the occurrence of symptoms and illnesses in the sample. The secondary objective of the study was to determine the relationship between work related factors and respiratory symptom and illnesses. Logistic regression was done to determine unadjusted and adjusted relationship between the independent (length of job years, type of work) and dependent variables (Chronic cough, chronic phlegm, wheeze, Chronic Bronchitis and Asthma). Both the independent variables were adjusted for age, smoking and education. Odds ratios with their confidence intervals were obtained of the different categories of independent variables.

## Results

The mean age of the workers was 31.03 years (SD= 9.0 years) and majority of them were uneducated (66.8%). 42.9% had ever smoked more than 1 cigarette a day for one year while 36.8% among them were current smokers (Table 1).

**Table 1:** Socio-demographic characteristics of adult male brick kiln workers in the outskirts of districts Dadu and Larkana, Sindh, Pakista (n=340)

**Variable**	**n (%)**
Age (years) *Mean (SD): 31.03(9.09)*	
18-34	227 (66.7%)
35-44	74 (21.8%)
45 and above	39 (11.5%)
Family size *Mean (SD): 7.70 (3.08)*	
Education Status	
Illiterate	232 (68.2%)
Primary	86 (25.3%)
Matriculate	16 (4.7%)
Intermediate	4 (1.2%)
Madresah education	2 (0.6%)
Monthly Income (PKR)	
*Mean (SD): 7864.7 (3344.2)*	
Type of work at brick Kiln	
Carriage and Placement	76 (22.4%)
Molding	195 (57.4%)
Baking	69 (20.2%)
Hours of work per *day*	
*Mean (SD)=8.28 (1.22)*	
Smoking Status	
Non- smoker	194 (57.1%)
Ever smoker	146 (42.9%)
Current smoker	125 (36.8%)
Characteristics of Smokers	
Age at Starting Smoking	
*Mean (SD)=19.84 (4.92)*	
Cigarettes per day	
*Mean (SD)=18.08 (7.74)*	

Almost one third of the workers (33.5%) coughed 4–6 times a day at the time of survey among whom 22.4% met the criteria of having chronic cough. 30.9% workers expectorated phlegm at least twice a day at the time of survey. Among them, 21.2% met the criteria of having chronic phlegm. 19.4% workers reported experiencing wheeze on exposure to smoke. Among them, 13.8% had two or more attacks of shortness of breath with wheezing in the past two months with normal breathing in between episodes of shortness of breath. One third (34.4%) reported mild Grade 1 dyspnea, 11.8% had Grade 2 dyspnea, 7.6% suffered from Grade 3 dyspnea while only 1.5% reported Severe Grade 4 dyspnea. 17.1% met the criteria of suffering from Chronic Bronchitis while 8.2% reported having been diagnosed by doctor as an asthmatic.

**Figure 1:**
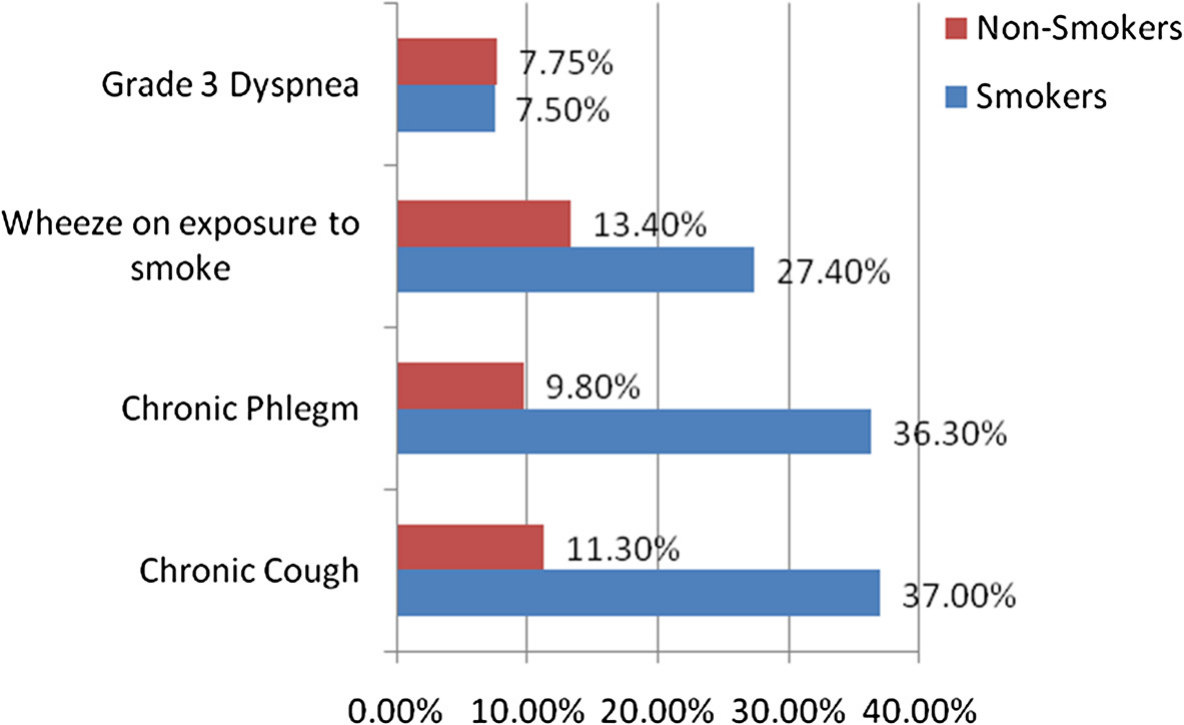
Frequency of Chronic Respiratory symptoms in non-smoking adult male brick kiln workers in the outskirts of districts Dadu and Larkana (n=194).

Figure 1 shows that smokers had more respiratory symptoms than non-smokers for all symptoms except dyspnea. The frequency was highest for chronic cough among smokers, while wheeze on exposure to smoke was highest among non-smokers.

Table 2 shows the Logistic Regression Analysis on association of occupational factors with chronic respiratory symptoms adjusted for age, smoking and education. Workers involved in molding were more likely to have chronic cough (OR=2.8, 95% CI 1.1-6.8, p≤0.05) and chronic phlegm (OR=3.8, 95% CI 1.4-10.3, p≤0.01) as compared to workers involved in carriage work. Stronger association was observed for involvement in brick baking with chronic cough (OR=3.8, 95% CI 1.4-10.3, p≤0.05) and chronic phlegm (OR=4.1, 95% CI 1.3-12.4, p≤0.01).

**Table 2:** Regression analysis on relationship of independent variables with Chronic Cough, Chronic Phlegm and Chronic Wheeze

	**Chronic cough**		**Chronic Phlegm**		**Chronic wheeze**	
	**OR (95% CI)**		**OR (95% CI)**		**OR (95% CI)**	
	Unadjusted	Adjusted#	Unadjusted	Adjusted#	Unadjusted	Adjusted#
Type of Work						
*Carriage*	1.00	1.00	1.00	1.00	1.00	1.00
*Baking*	4.3(1.6-10.9)^**^	3.8(1.4-10.3)^**^	5.3 (1.8-15.4) ^**^	4.1 (1.3-12.4)^*^	2.4 (0.86-6.95)	2.8 (0.8-10.0)
*Molding*	3.2 (1.3-7.4)^**^	2.8 (1.1-6.8)^*^	3.2 (1.76-12.15) ^**^	3.8 (1.4-10.3)^**^	2.2 (0.91-2.72)	3.0 (0.9-9.1)
Years of Work						
*5-9 years*	1.00	1.00	1.00	1.00	1.00	1.00
*10 and above*	2.7 (1.6-4.7)^***^	1.7 (0.9-3.3)	1.8 (1.08-3.10)^*^	1.1 (0.5-2.1)	4.4 (2.25-8.94) ^***^	2.2 (1.1-5.0)^*^

#Adjusted for age, smoking status and education.

*p<0.05 **p<0.01 ***p<0.001.

Length of job did not show any significant association with the symptoms.

Table 3 shows the Logistic Regression analysis on association of occupational factors with respiratory illnesses i-e Chronic Bronchitis and asthma. The multivariate model showed significant impact of brick baking on Chronic Bronchitis (OR= 3.7, 95% CI 1.1-11.6, p≤0.05) and Asthma (OR= 3.9, 95% CI 1.01-15.5, p≤0.05). Years of work >10 years did not show any significant association with both the outcomes after adjustment with the confounders.

**Table 3:** Regression analysis on relationship of independent variables with Chronic Bronchitis and Asthma

	**Chronic Bronchitis**		**Asthma**	
	**OR (95% CI)**		**OR (95% CI)**	
	**Unadjusted**	**Adjusted#**	**Unadjusted**	**Adjusted#**
Type of Work				
*Carriage*	1.00	1.00	1.00	1.00
*Baking*	4.2 (1.4-12.4)^*^	3.7 (1.1-11.6)^*^	4.1 (1.08-15.67)^*^	3.9 (1.01-15.5)^*^
*Molding*	3.3 (1.2-8.8)^*^	2.8 (1.0-8.0) ^*^	2.0 (0.57-7.21)	1.8 (0.4-6.5)
Years of Work				
*5-9 years*	1.00	1.00	1.00	1.00
*10 and above*	2.1 (1.2-3.8)^*^	1.06 (0.5-2.1)	1.87 (1.05-4.13)^*^	1.1 (0.4-3.0)

# Adjusted for age, smoking status and education.

^*^P=<0.05, ^**^P=<0.01, ^***^P=<0.001.

## Discussion

Most of the findings are similar to the results of the literature available on prevalence of respiratory symptoms and illnesses in different occupational settings where dust and smoke exposures are common. The results of the study show that 22.4% had chronic cough and 21.2% met the criteria of having chronic phlegm. 19.4% workers reported experiencing wheeze on exposure to smoke. A slightly higher frequency of chronic cough (31.8%) and phlegm (26.2%) was observed in the study from Croatia on brick kiln workers. Possible explanation for these high frequencies of respiratory symptom in brick kiln workers is higher exposure to air pollutants. The estimates of the symptoms mentioned above are also quite similar to the previous studies done on workers exposed to dust and smoke in other occupations [11-14].

17.1% of the workers in this study reported to be suffering from Chronic Bronchitis. It is a well established fact that smoking is a major risk factor for chronic bronchitis, however, 25-45% of these patients may not have history of smoking [15]. Therefore, a separate analysis was done on non-smoking brick kiln workers to relate the Chronic Bronchitis with occupational exposures to dust and smoke. 24% of Chronic Bronchitis in all the workers was contributed by non smoking individuals which is a strong evidence of the occupational effect on the respiratory illness. 8.2% reported having been diagnosed by doctor as an Asthmatic in this study. Prevalence of Asthma has ranged from 6 to 14% in other studies done in occupational settings where dust and smoke exposures are common [16].

Older age group of greater than 45 years group was also more than 3 times likely to develop Chronic Bronchitis and asthma. These findings are understandable as lung function starts declining progressively after 20–25 years [17]. None of the outcome variables were significantly associated with exposure of >10 years at workplace in the multivariate model. Stronger association was observed for workers involved in baking relative to carriage and placement workers as they were exposed to burning of coal and biomass smoke. Many studies that have found association of exposure to solid fuel smoke with Chronic Bronchitis [18]. As expected, strongest effect for Chronic Bronchitis was observed between all outcomes and smoking which is an established risk factor proved by many studies [19]. However, the findings of the study showed no significant association of smoking with asthma in both the models. A review published on the subject of smoking and Occupational Asthma confirms that relationship between them is complex and contradictory. The data from multiple occupations published over a period of 35 years in that review have shown that there is very little to support the view that the risk of occupational Asthma is increased in workers who are smokers [20]. Nonetheless, several studies have shown positive association of smoking with Asthma [21,22].

This is the first study of its kind to study the respiratory health of brick kiln workers. Standard validated questionnaire of American Thoracic Society was used to evaluate the respiratory symptoms and illnesses. However, due to limited resources; there are a few unavoidable limitations to this study. First, the findings of this study are based on subjective inquiry and are not supported by any objective measures. Due to limited resources and budget of the study, spirometry could not be performed to assess the lung functions. Second, due to limited scale of the study, no control group was included for comparison. However, comparisons have been made with previous findings to assess whether frequency of respiratory symptoms and illnesses was higher in these workers in comparison with general population or not. Third, although this study explains few factors associated with respiratory symptoms and illnesses, some factors could not be studied because we could include only a few numbers of brick kilns within the catchment area due to limited travel expenses. The districts in which this study was conducted, only males work at brick kilns due to cultural reasons; therefore, we could not study the impact on women. Only Bull trench kilns were covered in the survey which did not allow us to compare the findings with workers involved in brick kilns fuelled by gas with large chimneys for safe exit of smoke. Fourth, exposure measurements were also not possible due to financial constraints.

## Conclusion

A high frequency of respiratory symptoms and illnesses was observed in the brick kiln workers. Age, nature of work and smoking were strong predictors of developing these symptoms and illnesses. The evidence generated by this study needs to be further strengthened by conducting a more objective research on a large scale. Moreover, besides respiratory problems, other health risks like injuries and musculoskeletal problems should also be studied. The working conditions and their impact on the quality of life of the workers should also be explored. The fact that respiratory illnesses lead to compromised work output should also be assessed.

## Abbreviations

COPD: Chronic obstructive pulmonary diseases; ATS-DLD: American thoracic society – division of lung disease.

## Competing interest

Authors have no competing interests to declare.

## Authors’ contribution

SS was involved in literature search and proposal development for the study. He also contributed during the phase of data collection, data editing, analysis and its interpretation. He took the lead in developing this manuscript. AAN was involved in the initial process conceptualization and development of study proposal. He also supervised the data collection process and the write up of this manuscript. VK, AAJ, AMA and AY were involved in literature search and proposal development of the study. They participated actively in the data collection process and also contributed in data entry and write up of manuscript. All authors read and approved the final manuscript.

## Pre-publication history

The pre-publication history for this paper can be accessed here:

http://www.biomedcentral.com/1471-2458/12/999/prepub
